# Upregulation of miR-335-5p Contributes to Right Ventricular Remodeling via Calumenin in Pulmonary Arterial Hypertension

**DOI:** 10.1155/2022/9294148

**Published:** 2022-10-04

**Authors:** Hong Ma, Peng Ye, Ai-kai Zhang, Wan-de Yu, Song Lin, Ya-guo Zheng

**Affiliations:** ^1^Department of Cardiology, First Affiliated Hospital of Nanjing Medical University, Nanjing, Jiangsu 210029, China; ^2^Department of Cardiology, Nanjing First Hospital, Nanjing Medical University, Nanjing, Jiangsu 210008, China

## Abstract

Right ventricular (RV) failure determines the prognosis in pulmonary arterial hypertension (PAH), but the underlying mechanism is still unclear. Growing evidence has shown that microRNAs participate in RV remodeling. This study is undertaken to explore the role of miR-335-5p in regulating RV remodeling induced by PAH. Two PAH models were used in the study, including the monocrotaline rat model and hypoxia/su5416 mouse model. miRNA sequencing and RT-qPCR validation identified that miR-335-5p was elevated in the RV of PAH rats. In vitro, miR-335-5p expression was increased after angiotensin II treatment, and miR-335-5p inhibition relieved angiotensin II-induced cardiomyocyte hypertrophy. The luciferase reporter assay showed that calumenin was a target gene for miR-335-5p. Pretreatment with miR-335-5p inhibitors could rescue calumenin downregulation induced by angiotensin II in H9C2 cells. Moreover, intracellular Ca^2+^ concentration and apoptosis were increased after angiotensin II treatment, and miR-335-5p inhibition decreased intracellular Ca^2+^ accumulation and apoptosis. Finally, in vivo miR-335-5p downregulation (antagomir miR-335-5p) attenuated RV remodeling and rescued calumenin downregulation under conditions of hypoxia/su5416 exposure. Our work highlights the role of miR-335-5p and calumenin in RV remodeling and may lead to the development of novel therapeutic strategies for right heart failure.

## 1. Introduction

Pulmonary arterial hypertension (PAH) is a progressive lethal disease which is manifested as right heart failure and premature death [1]. Right ventricular (RV) adaptation and failure dramatically impact the prognosis of PAH [2]. The right ventricle exposed to chronic pressure overload adaptively exhibits concentric hypertrophy and maintains normal cardiac output. However, if the pathologic condition persists, it transits to maladaptive hypertrophy, presented as excessive fibrosis, decreased cardiac output, and right heart failure [3]. Current PAH therapies focus on pulmonary vascular remodeling; no RV-specific therapies exist. Therefore, exploring the underlying mechanisms of RV remodeling is urgent.

MicroRNAs (miRNAs) are a class of small noncoding ribonucleic acids which regulate gene expression at post-transcriptional level. Several studies have demonstrated that miRNAs are etiologically implicated in RV remodeling [4, 5]. They exhibit essential roles in hypertrophy and apoptosis of cardiomyocytes, proliferation, and migration of fibroblasts as well as angiogenesis of cardiac microvascular endothelial cells. Several miRNAs have been reported [6–9], including upregulated miR-21, miR-214, miR-199a, and miR-199b and downregulated miR-126, miR-223, miR-208, and miR-34. In the present study, through the use of RNA sequencing, we found that miR-335-5p was significantly increased in the RV of PAH rats, indicating that miR-335-5p may be implicated in RV hypertrophy and fibrosis induced by PAH.

miR-335-5p is a regulated intronic miRNA which is encoded by the second intron of the mesoderm-specific transcript gene. It has been related to certain types of cancer and participates in many oncogenic signaling pathways [10, 11]. It is a tumor suppressor gene and inhibits tumor cell proliferation, migration, and invasion. miR-335-5p was highly expressed in cardiomyocytes, and it was involved in cardiac differentiation through regulating mesoderm and progenitor marker expression via WNT and TGF-*β* signaling pathways [12]. Bioinformatics analysis also suggested that miR-335-5p might be associated with left ventricular remodeling after myocardial infarction [13]. But the role of miR-335-5p in RV remodeling remains unclear.

In the present study, we noted that miR-335-5p was significantly increased in the RV at 4 weeks following monocrotaline injection. Meanwhile, miR-335-5p expression was upregulated in angiotensin II-induced cardiomyocyte hypertrophy, whereas knockdown of miR-335-5p suppressed these responses. In addition, calumenin (CALU) was shown as a target of miR-335-5p. In the hypoxia/su5416 mouse model, in vivo miR-335-5p downregulation with antagomir miR-335-5p attenuated PAH-induced RV hypertrophy and fibrosis. Collectively, our findings indicated that miR-335-5p promoted RV remodeling, and therefore inhibition of miR-335-5p might be useful for the treatment of right heart failure induced by PAH.

## 2. Materials and Methods

### 2.1. Animal Models

Animals were supplied by the animal center of Nanjing Medical University. We followed the guide for the care and use of laboratory animals, and all the experimental protocols were permitted by the institutional animal care and use committee of Nanjing Medical University. Two PAH models were used in the study, including the monocrotaline (MCT) rat model and hypoxia/su5416 mouse model. Sixteen adult male SD rats at 6 weeks were randomly divided into two groups: the control group (*n* = 8) and the PAH group (*n* = 8). Monocrotaline (60 mg/kg, Sigma, St Louis, MO) and vehicle were intraperitoneally injected in each group, respectively. After 28 days, the rats underwent right heart catheterization and echocardiographic assessments.

Hypoxia/su5416 mouse model was established according to our previous study [14]. Male C57/BL6 mice at 8 weeks were subcutaneously injected with 20 mg/kg su5416 and then exposed to chronic hypoxia (10% O_2_) for the subsequent 4 weeks. Twenty-four mice were randomly divided into three groups (*n* = 8): normoxia control, hypoxia+antagomir NC, and hypoxia+antagomir-335-5p. Antagomir specific for miR-335-5p and a non-specific negative control (NC) were designed as described [15] and synthesized by GenePharma Co., Ltd (Shanghai, China). The sequence of antagomir-335-5p was 5′-mA(s)mC(s)mAmUmUmUmUmUmCmGmUmUmAmUmUmGmCmUmCmU(s)mU(s)mG(s) mA (s)-Chol-3′. The antagomir NC group and antagomir-335-5p group were injected, respectively, via the tail vein with NC and antagomir-335-5p oligonucleotides at 80 mg/kg for 3 consecutive days. After the injection of oligonucleotides, the mice were placed in the hypoxia chamber. Hemodynamic and echocardiographic assessments were performed at day 28 after hypoxia exposure.

### 2.2. Hemodynamic Measurements

Right heart catheterization was performed following our previous published methods [14, 16]. For rats, the polyethylene catheter was inserted into the main pulmonary artery through the right internal jugular vein to acquire mean pulmonary arterial pressure (mPAP). As the catheter could not get into the pulmonary artery for the mice, only right ventricular systolic pressure (RVSP) was measured in the hypoxia/su5416 model. After catheterization, all the animals were sacrificed, and the heart and lung tissues were harvested. The right ventricle (RV) was separated from the left ventricle plus ventricular septum (LV + S) and weighted, respectively. The ratio of RV/(LV + S) was calculated as the right ventricular hypertrophy index (RVHI).

### 2.3. Echocardiographic Assessments

Echocardiography was performed as previously described [17]. After anesthesia, the Vevo 2100 imaging system (Visual Sonics, Toronto, Canada) was used to obtain the echocardiographic images. The following parameters were assessed including RV free wall thickness (RVWT) from the parasternal long-axis view, RV internal diameter, and tricuspid annular plane systolic excursion (TAPSE) from the apical four-chamber view. We also measured pulmonary artery acceleration time (PAAT) and pulmonary artery ejection time (PAET). The PAAT/PAET ratio was then calculated as an indirect measure of systolic PAP.

### 2.4. Morphologic Analysis

The lung and heart were fixed in 4% paraformaldehyde solution for light microscopy or 2% glutaraldehyde for transmission electron microscopy. The tissues were dehydrated and embedded in paraffin, and then cut into 5 *μ*m slices for hematoxylin-eosin (HE) and Masson stain. Images were acquired using a light microscope (Nikon, Tokyo, Japan).

### 2.5. High-Throughput RNA Sequencing (RNA-Seq)

Total RNA was extracted from three randomly selected right ventricle tissue samples using Trizol (Invitrogen, CA, USA). Sequencing libraries were prepared with NEBNext® Multiplex Small RNA Library Prep Set for Illumina and sequenced using the Illumina Hiseq 2500/2000 platform. After quality evaluation (FastQC) and data filtering (Cutadapt), the small RNA tags were mapped to a reference sequence using Bowtie without mismatch, to analyze their expression and distribution on the reference sequences. miRNA expression levels were quantified in TPM (transcript per million). DESeq R package was used to analyze the differential expression of miRNA levels between the two groups. The *P* values were then corrected with the Benjamini-Hochberg method.

### 2.6. Cell Culture

H9C2 cells from ATCC were maintained in Dulbecco's modified Eagle's medium (DMEM) which is supplemented with 10% fetal bovine serum. When the cell lines reached 80% confluence, they were digested with 0.05% trypsin-EDTA and plated in a 6-well plate with 3 × 10^5^ cells per well. Next day, the cells were transfected with miR-335-5p inhibitor (5′-ACAUUUUUCGUUAUUGCUCUUGA-3′) or scramble control (5′-CAGUACUUUUGUGUAGUACAA-3′) (GenePharma, Shanghai, China) for 6 hours and then incubated with angiotensin II (0.1 *μ*mol/L, Sigma) to induce cardiomyocyte hypertrophy as described previously [18]. After 48 hours, cells were harvested for gene and protein expression analysis.

### 2.7. Luciferase Assay

Three different algorithms (TargetScan, miRanda, and PicTar) were used to predict target gene of miR-335-5p. All three algorithms suggested that calumenin (CALU) was the downstream target of miR-335-5p. The segments of CALU 3′-UTR containing the putative miR-335-5p binding sites were PCR-amplified, and the amplification product was inserted into the pGL3 vector, resulting in the wild-type pGL3-CALU-3′UTR. The corresponding mutant vectors were formed by using mutated calumenin sequence. HEK293 cells were co-transfected with wild-type and mutated pGL3-CALU-3′UTR with miR-335-5p mimics or negative control, respectively. At 48 h after transfection, the cells were harvested, and dual-luciferase reporter assay kit was used to determine luciferase activity.

### 2.8. Immunohistochemical Staining and Immunofluorescence

After deparaffinizing, heat-mediated antigen retrieval was achieved by incubating sections in sodium citrate buffer (pH 6.0) for 20 minutes. Then, the slices were treated with 3% hydrogen peroxide to quench endogenous peroxidase activity and successively incubated with the primary antibody (rabbit anti-calumenin monoclonal antibody from Abcam Corporation) at 4 °C overnight. After washing three times, the sections were subsequently incubated with biotinylated secondary antibody (Zhongshan Jinqiao Biotechnology) for 30 minutes at room temperature. Finally, diaminobenzidine was added, and the brown color indicated a positive reaction.

H9C2 cells were stained with antibodies against *ɑ*-SMA (Sigma, St. Louis, MO) to measure cardiomyocytes size. After permeabilization with 0.3% Triton X-100 for 10 minutes, non-specific sites were blocked with 1% bovine serum albumin (BSA) for 1 hour. Primary antibody to *ɑ*-SMA actin was applied at 4 °C overnight. Then, the cells were incubated with the respective secondary antibody at room temperature for 1 hour and followed by DAPI counterstaining. Images were acquired using laser scanning confocal microscopy (LSM 710; Carl Zeiss, Germany).

### 2.9. Real-Time Quantitative Polymerase Chain Reaction (RT-qPCR)

Total RNA was isolated from the heart tissues and H9C2 cells, and reverse transcription was performed according to the instructions. qPCR was carried out using SYBR Premix Master Mix (Thermo Fisher Scientific Inc., Shanghai, China). The primer sequences were listed in Supplemental Table 1. miRNA analysis was performed using TaqMan MicroRNA Assay (GenePharma, Shanghai, China), and U6 was used as a reference gene.

### 2.10. Western Blotting

Total protein was extracted from the heart tissues and H9C2 cells. After protein concentration measurement, these proteins were separated by SDS-PAGE and subsequently transferred to PVDF membranes. Then, the filter membranes were incubated overnight at 4 °C with the primary antibody (Calumenin from Abcam, Collagen I and Collagen III from Cell Signaling). The membranes were further incubated with corresponding secondary antibody for two hours at room temperature and detected using the enhanced chemiluminescence (ECL) system. *β*-Actin was used as a loading control. The bands were analyzed by the quantity one.

### 2.11. TUNEL Staining

TUNEL Apoptosis Assay Kit (Beyotime Biotechnology, Beijing, China) was used to detect the apoptotic cells. The nuclei were counterstained with DAPI. The positive cells were characterized by green nuclei. The imagines were observed with LSM710 confocal laser scanning microscope (LSM 710; Carl Zeiss, Germany). The cell apoptotic rate was calculated as the percentage of TUNEL-positive cells.

### 2.12. Intracellular Ca^2+^ Concentration Measurements

H9C2 cardiomyocytes were incubated with Fluo-3/AM (Beyotime Biotechnology, Beijing, China) at 37 °C for 60 min based on the instructions. The Ca^2+^ Fluorescence was monitored at 488 nm with LSM710 confocal laser scanning microscope (LSM 710; Carl Zeiss, Germany).

### 2.13. Statistical Analysis

All continuous variables are expressed as mean ± SD (standard deviation). Student's *t* test was used to determine the statistical difference between the two groups. Differences between multiple groups were compared with ANOVA followed by Bonferroni's post hoc test. All analyses were performed using SPSS version 22.0 (SPSS Inc., Chicago, IL). *p* value < 0.05 was considered statistically significant.

## 3. Results

### 3.1. Right Ventricle Remodeling in MCT-Induced PAH

**Figure 1:**
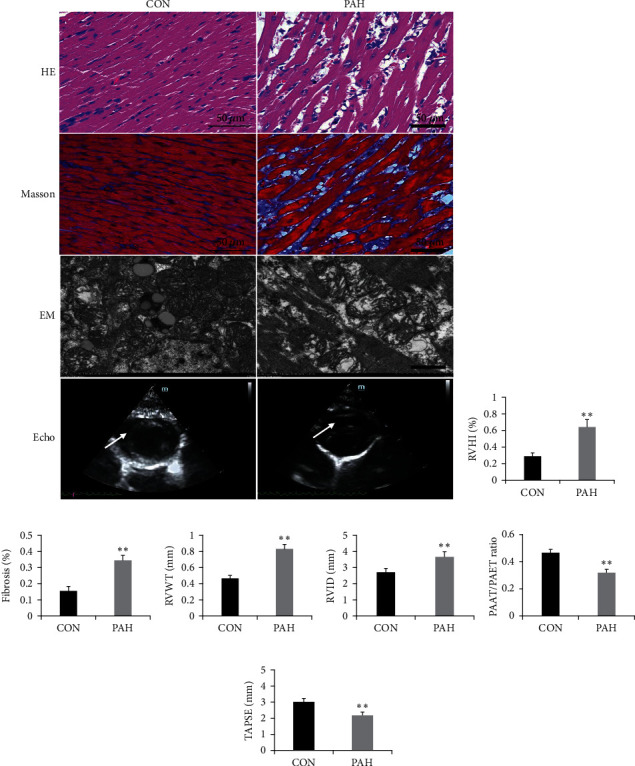
Right ventricle remodeling in MCT-induced PAH. (a) Representative images of HE, Masson staining, electron microscopy, and echocardiography in right ventricle of PAH rats; scale bars indicate 50 *μ*m or 10 *μ*m; (b) RVHI changes after MCT injection; (c) fibrosis changes after MCT injection; (d) RVWT changes after MCT injection; (e) RVID changes after MCT injection; (f) PAAT/PAET ratio changes after MCT injection; and (g) TAPSE changes after MCT injection. Arrow indicates right ventricular hypertrophy and mitochondrial swelling in PAH group. EM: electron microscopy; RVHI: right ventricular internal diameter; RVWT: right ventricular wall thickness; RVID: right ventricular internal diameter; TAPSE: tricuspid annular plane systolic excursion; PAAT: pulmonary artery acceleration time; PAET: pulmonary artery ejection time; mPAP: mean pulmonary arterial pressure. Data are presented as mean ± SD; *n* = 8 per group; ^∗^*P* < 0.05 and ^∗∗^*P* < 0.01.

According to our previous study [16], we established the MCT-induced PAH model. PAH rats displayed significant right ventricular hypertrophy and dysfunction and manifested as decreased TAPSE and increased RVHI, RVWT, and RVID (Figures 1(b), 1(d), 1(e), and 1(g)). HE staining suggested myocyte hypertrophy, focal myolysis, cellular necrosis, and vacuolar degeneration (Figure 1(a)). Moreover, apparent right ventricular fibrosis was also observed in PAH rats (Figure 1(c)). Electron microscopy revealed that there were significant functional mitochondrial changes in right ventricle, characterized by mitochondrial swelling and decreased matrix density (Figure 1(a)).

### 3.2. High-Throughput RNA Sequencing

**Figure 2:**
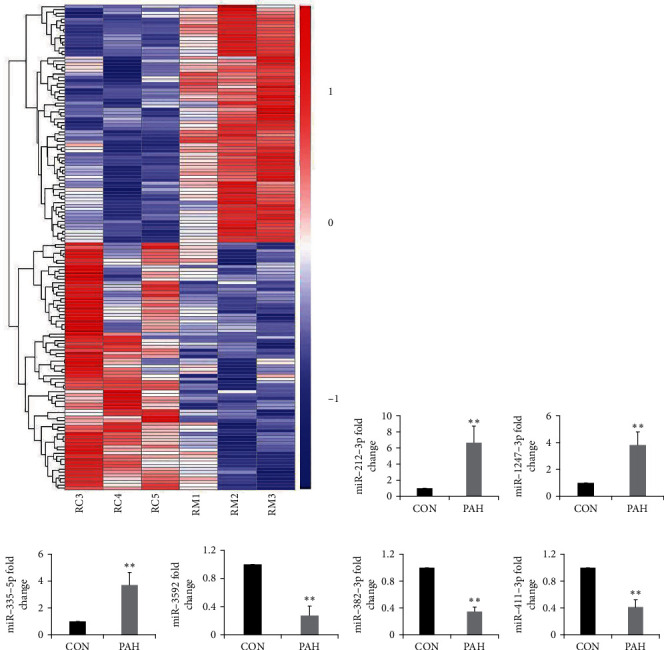
High-throughput RNA sequencing. (a) The distributions of the total expression of the miRNAs in six samples (*n* = 3 per group). (b-g) RT-qPCR validation of six dysregulated miRNAs in PAH rats compared with the controls. Data are presented as mean ± SD; *n* = 4 per group; ^∗^*P* < 0.05 and ^∗∗^*P* < 0.01.

RNA-Seq results for six right ventricular tissues (*n* = 3 in each group) were used for a comprehensive analysis. In total, 151 miRNAs (74 upregulated and 77 downregulated) were differentially expressed in PAH rats compared with controls (Figure 2(a)). The top 10 upregulated and downregulated miRNAs are shown in Supplementary Table 2. To verify the reliability of the sequencing results, we selected three upregulated and three downregulated miRNAs for RT-qPCR. The result suggested that miR-212-3p, miR-1247-3p, and miR-335-5p were significantly upregulated, while miR-3592, miR-382-3p, and miR-411-3p were significantly downregulated, in PAH rats compared with the controls (Figure 2(b)). These findings were consistent with the RNA-Seq data, potently validating their reliability. miR-335-5p was highly expressed in cardiomyocytes. Previous studies have demonstrated that miR-335-5p was involved in cardiac differentiation and left ventricular remodeling after myocardial infarction [12, 13]. Therefore, we chose miR-335-5p for further study.

### 3.3. Upregulated miR-335-5p in Angiotensin II-Induced Cardiomyocyte Hypertrophy

**Figure 3:**
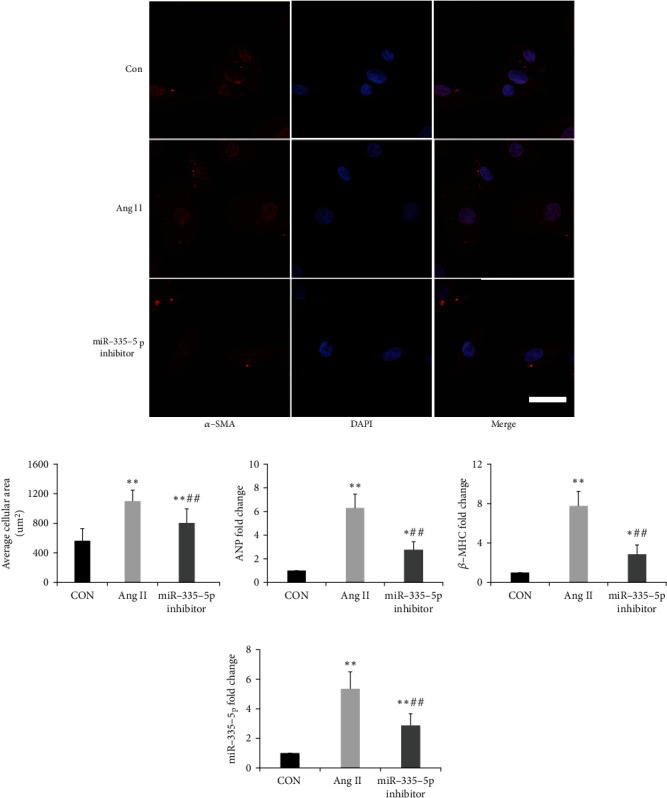
Upregulated miR-335-5p in Ang II-induced cardiomyocyte hypertrophy. (a) Representative immunofluorescence micrographs of H9C2 cells stained with *ɑ*-SMA antibody; scale bars indicate 20 *μ*m; (b) quantitation of average cell surface area; and (c-e) ANP, *β*-MHC, and miR-335-5p fold changes in cardiomyocytes treated with control, Ang II, and Ang II + miR-335-5p inhibitor. Ang II: angiotensin II. Data are presented as mean ± SD; *n* = 4 per group; ^∗^*P* < 0.05 and ^∗∗^*P* < 0.01 versus the control group; ^#^*P* < 0.05 and ^##^*P* < 0.01 versus the Ang II group.

The cell surface area was significantly increased in angiotensin II-induced cardiomyocyte hypertrophy. Compared to the control, miR-335-5p levels were significantly increased in angiotensin II-induced cardiomyocyte hypertrophy (Figure 3(e)). Pretreatment with miR-335-5p inhibitors could decrease the cell surface area induced by angiotensin II (Figures 3(a) and 3(b)). Similarly, miR-335-5p inhibition could also decrease the expression of ANP and *β*-MHC in in angiotensin II-induced cardiomyocyte hypertrophy (Figures 3(c) and 3(d)). Therefore, angiotensin II could facilitate miR-335-5p expression, and its pro-hypertrophy effects on cardiomyocytes were inhibited by pretreatment with miR-335-5p inhibitor.

### 3.4. miR-335-5p Targets Calumenin in Cardiomyocytes

**Figure 4:**
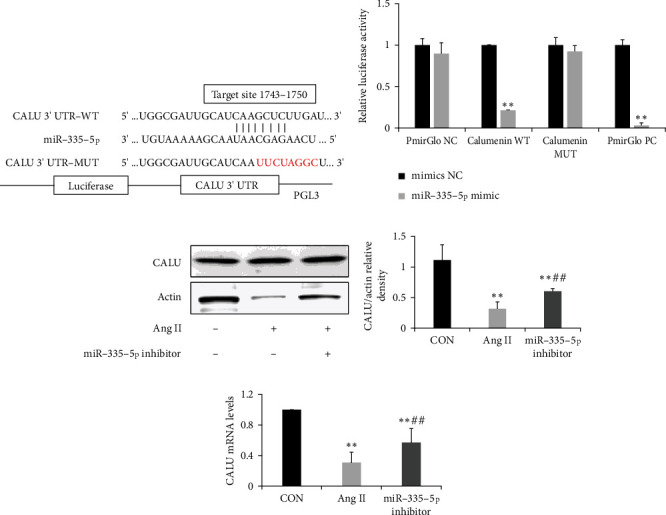
miR-335-5p targets calumenin in H9C2 cells. (a) Predicted binding sequences between miR-335-5p and 3′UTR of calumenin; (b) luciferase activities of the luciferase reporter vectors containing wild-type or mutant calumenin 3′UTR in 293 T cells co-transfected with miR-335-5p mimic or negative control (*n* = 3); (c-e) Representative blots and quantified data showing protein and mRNA expression levels of calumenin in H9C2 cells after being transfected with Ang II or miR-335-5p inhibitor (*n* = 4 per group). Ang II: Angiotensin II; CALU: calumenin; WT: wild type; and MUT: mutant. Data are presented as mean ± SD; ^∗^*P* < 0.05 and ^∗∗^*P* < 0.01 versus the control group; ^#^*P* < 0.05 and ^##^*P* < 0.01 versus the Ang II group.

We used three online databases, including Targetscan, miRanda, and PicTar, to predict the target gene of miR-335-5p. Combined with previous reported genes involved in cardiomyocyte hypertrophy, we focused on calumenin as a potential target. The luciferase assay was performed to evaluate if miR-335-5p overexpression affected the luciferase activity of different reporter vectors containing wild-type or mutant calumenin 3′UTR (Figures 4(a) and 4(b)). The result suggested that miR-335-5p overexpression could decrease luciferase activity of calumenin wide-type constructs, but not calumenin mutant constructs. Furthermore, the expression of calumenin was decreased in angiotensin II-induced cardiomyocyte hypertrophy, and pretreatment with miR-335-5p inhibitors could rescue calumenin downregulation in H9C2 cells (Figures 4(c), 4(d), and 4(e)).

**Figure 5:**
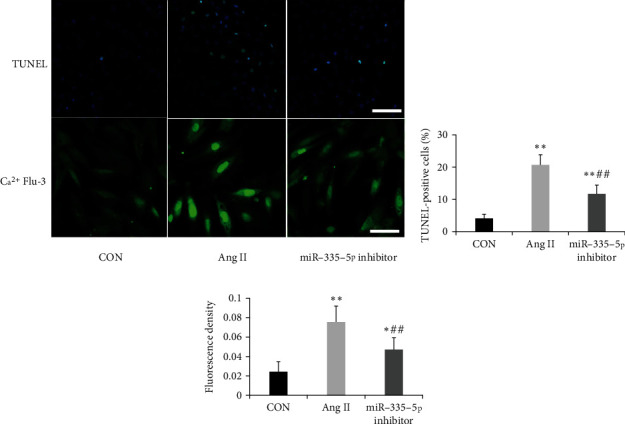
Suppression of miR-335-5p decreased apoptosis and Ca^2+^ accumulation in H9C2 cardiomyocytes induced by Ang II. (a) The apoptosis and intracellular Ca^2+^ accumulation of H9C2 cardiomyocytes were evaluated by TUNEL assay and fluo-3/AM determination; scale bars indicate 50 *μ*m; (b, c) quantified data showing the apoptotic rate and the intensity of Ca^2+^ fluorescence after being transfected with Ang II or miR-335-5p inhibitor (*n* = 5 per group). Data are presented as mean ± SD; ^∗^*P* < 0.05 and ^∗∗^*P* < 0.01 versus the control group; ^#^*P* < 0.05 and ^##^*P* < 0.01 versus the Ang II group.

Calumenin significantly alleviated endoplasmic reticulum (ER) stress and Ca^2+^ overload and thus inhibited cellular apoptosis in rat cardiomyocytes [19, 20]. The apoptosis and intracellular Ca^2+^ accumulation of H9C2 cardiomyocytes were evaluated by TUNEL assay and fluo-3/AM determination (Figure 5(a)). The result showed that the apoptotic rate and the intensity of Ca^2+^ fluorescence were significantly increased after angiotensin II treatment. However, pretreatment with miR-335-5p inhibitors could decrease the increase of apoptosis and intracellular Ca^2+^ accumulation (Figures 5(b) and 5(c)).

### 3.5. miR-335-5p Downregulation Alleviates Right Ventricular Remodeling in Hypoxia/su5416-Induced PAH

**Figure 6:**
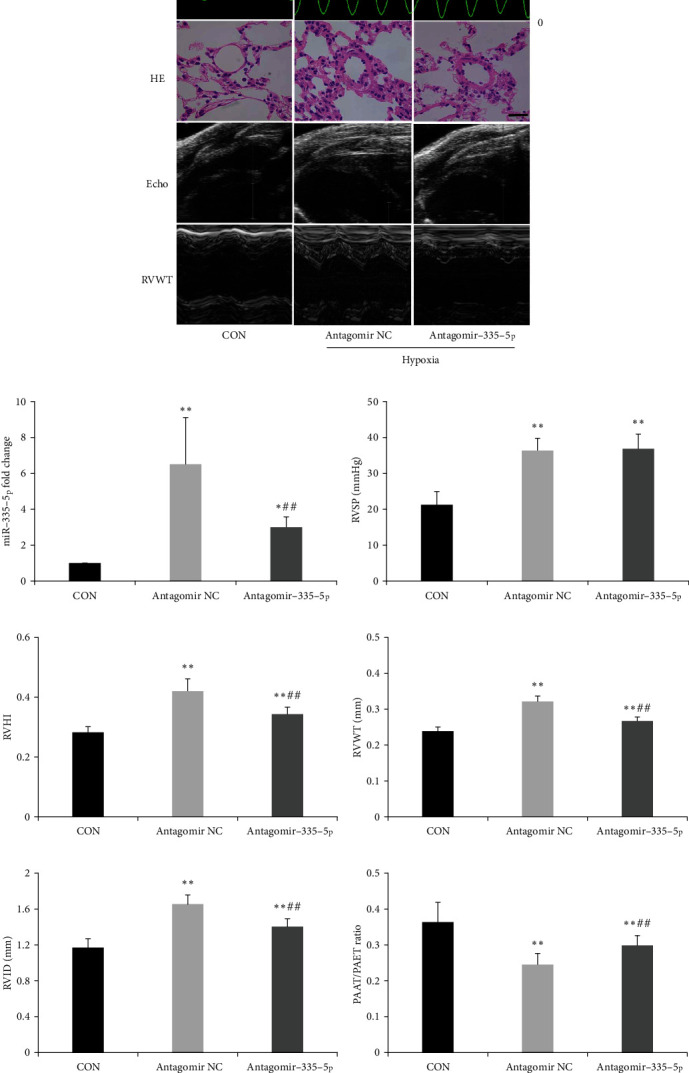
miR-335-5p downregulation alleviates right ventricular hypertrophy in hypoxia/su5416-induced PAH. (a) Representative images of RVSP, lung HE, and echocardiography in hypoxia/SU5416 mice, scale bars indicate 25 *μ*m. (b) Decreased miR-335-5p in the RV of mice treated with antagomiR-335-5p as determined by RT-qPCR. (c) Hemodynamic measurements revealed unchanged RVSP after antagomiR-335-5p treatment. (d) RVHI was alleviated after antagomiR-335-5p treatment. (e-g) RVWT, RVID, and PAAT/PAET ratio were measured by echocardiography in PAH mice treated with antagomiR-335-5p or NC. RVHI: right ventricular internal diameter; RVWT: right ventricular wall thickness; RVID: right ventricular internal diameter; PAAT: pulmonary artery acceleration time; PAET: pulmonary artery ejection time; RVSP: right ventricular systolic pressure. Data are presented as mean ± SD; *n* = 8 per group; ^∗^*P* < 0.05 and ^∗∗^*P* < 0.01 versus the control group; ^#^*P* < 0.05 and ^##^*P* < 0.01 versus the antagomir NC group.

To investigate the role of miR-335-5p on right ventricular dysfunction, miR-335-5p antagomir was injected via tail vein for 3 consecutive days prior to hypoxia/su5416 exposure. After 4 weeks, treatment with antagomiR-335-5p resulted in a significant reduction of miR-335-5p in the right ventricle (RV) (Figure 6(b)). Echocardiography revealed that RV dilatation and RV thickness were attenuated in antagomiR-335-5p-treated mice (Figures 6(e) and 6(f)). Moreover, antagomiR-335-5p administration could prevent PAH-induced increases in RV hypertrophy index (Figure 6(d)). Interestingly, RVSP and pulmonary vascular remodeling were unchanged between groups, indicating that in vivo knockdown of miR-335-5p had no effect on pulmonary histopathological changes (Figure 6(a)).

**Figure 7:**
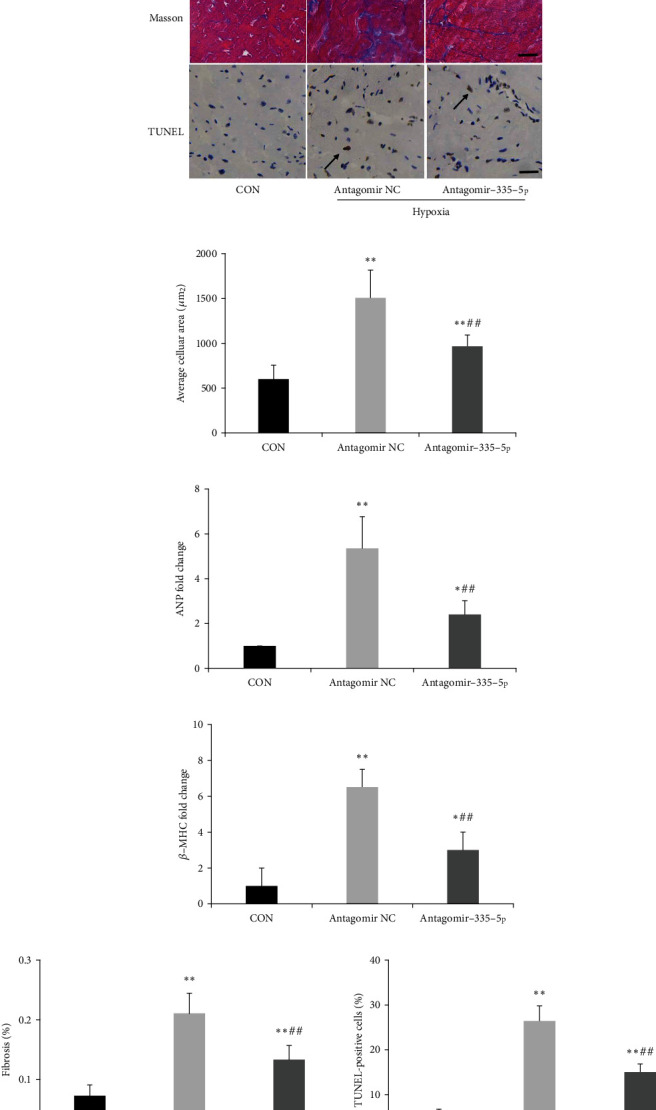
miR-335-5p downregulation alleviates fibrosis and apoptosis in the right ventricle. (a) Representative images of HE, Masson, and TUNNEL in the RV of hypoxia/su5416 mice; scale bars indicate 25 *μ*m. (b) Histological examination indicated that miR-335-5p inhibition reduced cardiomyocyte cross-sectional areas. (c) Decreased ANP and *β*-MHC in the RV of mice treated with antagomiR-335-5p as determined by RT-qPCR. (e) Masson staining indicated that miR-335-5p inhibition reduced fibrosis. (f) Quantified data showing the apoptotic rate after being treated with antagomiR-335-5p; Data are presented as mean ± SD; *n* = 6 per group; ^∗^*P* < 0.05 and ^∗∗^*P* < 0.01 versus the control group; ^#^*P* < 0.05 and ^##^*P* < 0.01 versus the antagomir NC group.

**Figure 8:**
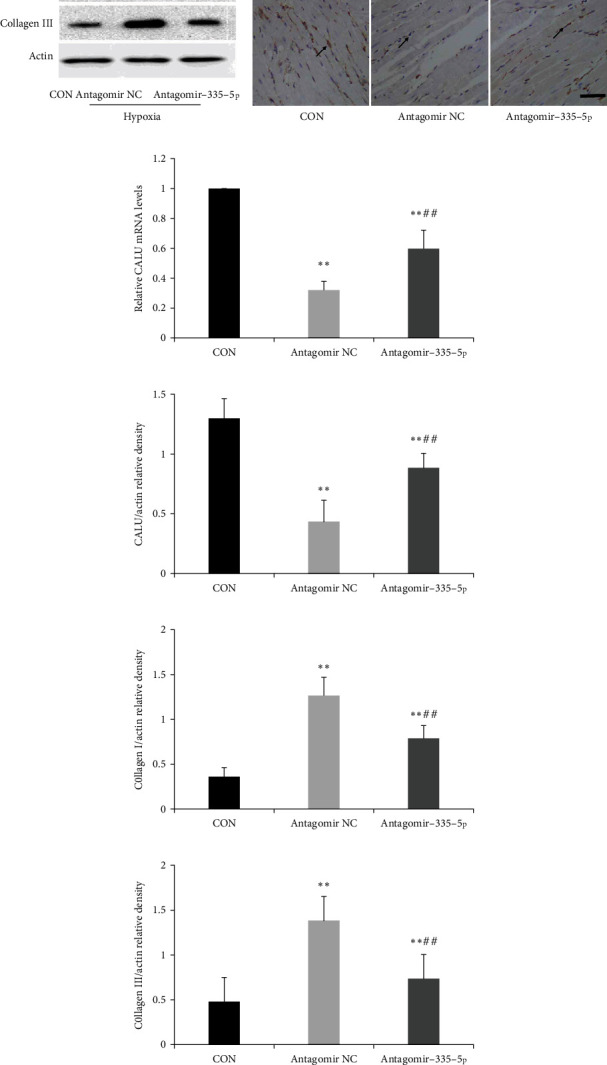
miR-335-5p inhibition rescues calumenin downregulation in hypoxia/su5416-induced PAH. (a) Representative immunoblots of calumenin, collagen I, and collagen III in hypoxia/su5416-induced PAH. (b) Immunohistochemical staining of calumenin in three groups; scale bars indicate 50 *μ*m. (c) RT-PCR revealed that miR-335-5p inhibition rescued the downregulation of calumenin induced by hypoxia/su5416 exposure. (d-f) Statistical analysis of relative density of calumenin, collagen I, and collagen III. Arrow indicates the representative staining of CALU in the RV. Data are presented as mean ± SD; *n* = 4/group; ^∗^*P* < 0.05 and ^∗∗^*P* < 0.01 versus the control group; ^#^*P* < 0.05 and ^##^*P* < 0.01 versus the antagomir NC group.

Histologic analysis also revealed that antagomiR-335-5p administration attenuated the enlargement in cardiomyocyte cross-sectional areas after hypoxia exposure (Figures 7(a) and 7(b)). Myocardial hypertrophy marker genes ANP and *β*-MHC were also decreased after miR-335-5p inhibition (Figures 7(c) and 7(d)). As cardiac fibrosis was critical in the progression of cardiac remodeling, we also quantified the extent of fibrosis by Masson staining. RV collagen deposition was obviously reduced in PAH mice treated with antagomiR-335-5p when compared to those treated with antagomir NC (Figures 7(a) and 7(e)). Moreover, TUNEL assay suggested that the apoptotic rate was significantly increased in the RV of PAH mice and miR-335-5p inhibition could decrease the increase of apoptosis (Figures 7(a) and 7(f)). Consistent with the results of Masson staining, myocardial fibrosis markers including collagen I and collagen III were upregulated in the right ventricle of PAH mice, and antagomiR-335-5p treatment could obviously reduce the expression of collagen I and III (Figures 8(a), 8(e), and 8(f)).

As calumenin is the target gene of miR-335-5p, we also investigated the changes of calumenin mRNA and protein levels. We found that calumenin was located in the cytoplasm of cardiomyocyte and calumenin expression was significantly decreased in PAH mice (Figures 8(a) and 8(b)). However, antagomiR-335-5p treatment could rescue the downregulation of calumenin induced by hypoxia/su5416 exposure (Figures 8(c) and 8(d)).

## 4. Discussion

Although the initial insult involves pulmonary vascular, RV function is the main determinant of prognosis in PAH [2]. When exposed to increased afterload, the RV transfers from initial adaptive transformation to subsequent maladaptive transformation, leading to adverse morphological changes and functional decline, which is defined as RV remodeling [4, 8]. The process is complex, and it depends not only on the severity of PAH, but also on changes in myocardial metabolism, neurohormonal activation, rate of myocardial hypertrophy and fibrosis, as well as genetic and epigenetic factors. This multifactorial interplay likely explains the variability of RV function among PAH patients.

Monocrotaline (MCT) rat model is a commonly used experimental model of RV failure [8]. Apart from clinical signs, including cachexia, dyspnea, ascites, and congestion, apparent RV enlargement, fibrosis, systolic dysfunction, and mitochondrial changes were also observed. Abnormal miRNA expression has been shown in this model. For example, miR-126 and miR-208 were found to be downregulated and miR-155 upregulated in MCT-induced PAH [8, 21]. Using miRNA sequencing, we identified 151 differentially expressed miRNAs in the RV of PAH rats. We focused on miR-335-5p as it was associated with cardiac fibrosis and hypertrophy. miR-335-5p participated in the pathogenesis of myocardial ischemia, and miR-335-5p overexpression alleviated myocardial ischemia reperfusion injury [22]. Kay et al. [12] found that miR-335-5p was involved in cardiac differentiation through inducing mesoderm and progenitor marker expression. Furthermore, miR-335-5p could also regulate angiotensin II-induced cardiac fibrosis and hypertrophy by targeting galectin-3 [23]. These studies suggested that miR-335-5p might act as a protective factor in cardiac fibrosis and hypertrophy.

We found miR-335-5p was obviously upregulated in the right ventricle of PAH rats. miR-335-5p levels were also increased in an in vitro model of cardiac hypertrophy, and inhibition of miR-335-5p attenuated angiotensin II-induced cardiomyocyte hypertrophy. Similarly, Goncalves et al. [24] have showed that miR-335-5p is upregulated in post myocardial infarction myocardium and angiotensin II stimulated H9C2 cells. Our findings indicated that miR-335-5p might be a pro-hypertrophic factor in right heart failure. This discrepancy can be explained by the complex tissue- and cell-based specific roles of miR-335-5p in right and left heart failure. Right ventricle (RV) is different from left ventricle (LV) in terms of embryologic origin, metabolism, vascularity, and the response to pressure overload [25]. There are interesting differences in microRNA expression between the RV and LV, and this difference is maintained during afterload stress. In a murine model of pulmonary artery constriction, Reddy et al. [9] have reported four RV-specific microRNAs which are not increased in LV hypertrophy. Moreover, miR-208a expression has been shown to be maintained in LV failure, while its levels decreased during RV failure, suggesting a chamber-specific regulatory mechanism [21, 26]. Therefore, the differential role of miR-335-5p in RV failure is a novel finding.

Antagomirs are widely used to antagonize endogenous miRNAs both in vivo and in vitro studies [27]. A long-lasting but reversible inhibition of miR-335-5p function was achieved after repeated intravenous systemic administration of antagomir-335-5p in a mouse model of PAH. Our results suggested that antagomir miR-335-5p effectively reduced PAH-induced right ventricular hypertrophy and fibrosis. We also found that miR-335-5p inhibition had no effect on right ventricular systolic pressure and pulmonary arteriole pathology, suggesting that the cardioprotective effect was independent of pulmonary vascular changes. RV failure was associated with myocardial apoptosis, and increasing myocardial apoptosis led to deteriorated cardiac function, therefore taking part in the pathological processes of RV remodeling [28, 29]. TUNEL staining indicated less cardiomyocyte apoptosis in antagomiR-335-5p group than in the NC group. Therefore, we supposed that miR-335-5p inhibition improved PAH-induced right ventricular remodeling through reducing cardiomyocyte apoptosis. Pharmacological modulation of miR-335-5p expression could be exploited for new therapeutic measures for RV remodeling in PAH.

We also identified that calumenin (CALU) was a target of miR-335-5p. Calumenin is an endoplasmic reticulum resident Ca^2+^-binding protein and acts as a molecular chaperone to maintain homeostasis of calcium cycling in mammalian hearts [30]. It has been involved in many pathophysiologic processes including vascular calcification, thrombosis, and cell apoptosis. Calumenin interacts with endoplasmic reticulum Ca^2+^-ATPase in rat cardiac ER, and its overexpression in rat neonatal cells showed decreased ER Ca^2+^ uptake and decreased fractional Ca^2+^ release [31, 32]. In neonatal rat ventricular cardiomyocytes, calumenin overexpression ameliorated ER stress and inhibited ER stress induced apoptosis [19]. Calumenin can also relieve ER stress-initiated apoptosis in viral myocarditis [20]. Recently, Zhang et al. [33] identified that calumenin was downregulated in dilated cardiomyopathy. In this study, our results revealed that calumenin levels were decreased in the RV of PAH mice and miR-335-5p antagomir could rescue the downregulation of calumenin, indicating that miR-335-5p might regulate RV remodeling via calumenin.

**Figure 9:**
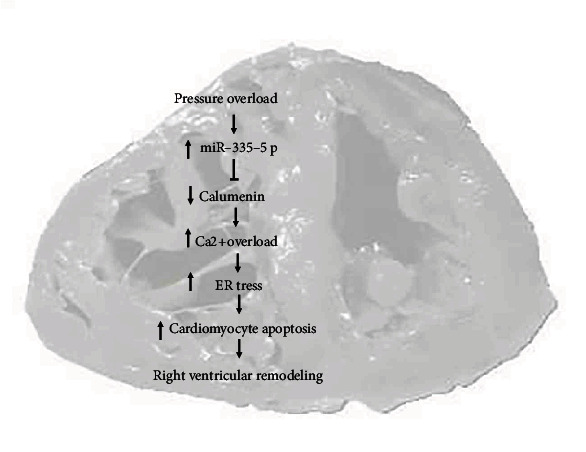
Schematic overview of the proposed mechanisms for miR-335-5p/calumenin signaling pathway in right ventricular remodeling. ER stress: endoplasmic reticulum stress.

Consistent with calumenin changes, we also found that intracellular Ca^2+^ concentration and apoptosis were increased after angiotensin II treatment, and miR-335-5p inhibition decreased intracellular Ca^2+^ accumulation and apoptosis. Therefore, we supposed that RV pressure overload induced by PAH increased cardiomyocyte expression of miR-335-5p, subsequently caused calumenin downregulation, leading to Ca^2+^ overload and ER stress, and finally contributed to cardiomyocyte apoptosis and right ventricular remodeling (Figure 9). But the direct relationship between calumenin and Ca^2+^ accumulation and their functional roles in cardiomyocyte apoptosis were not established and required further confirmation.

There are several limitations of this study. Firstly, H9C2 cells were used in this study, but it should be better to measure the effects in rat neonate cardiomyocytes. Secondly, fibroblast proliferation was also involved in right ventricular remodeling, but we did not measure the effects of miR-335-5p on RV fibroblast proliferation. Thirdly, we found that miR-335-5p downregulation caused less apoptosis and less calcium accumulation in angiotensin II induced cardiomyocyte hypertrophy. CALU was the target gene of miR-335-5p and had function in Ca^2+^ overload and cardiomyocyte apoptosis. But these experiments could not confirm the direct relationship between CALU/miR-335-5p/apoptosis. miR-335 downregulation could also lead to the reduction of apoptosis and Ca^2+^ accumulation through other factors. Therefore, further studies are still needed to confirm the relation of miR-335-5p/CALU and downstream phenotypes.

## 5. Conclusion

Our results revealed that miR-335-5p expression was elevated in the right ventricle of PAH. miR-335-5p inhibition exhibited protective effects on PAH-induced right ventricular remodeling through targeting calumenin, implying that miR-335-5p along with calumenin signaling might provide novel sight for a better understanding of RV remodeling. However, the role of miR-335-5p requires to be confirmed in future studies.

## Data Availability

The data used during the present study are available from the corresponding author upon reasonable request.

## Supplementary Materials

Supplementary 1 Supplementary Table 1. Primer sequences used for quantitative RT-qPCR.

Supplementary 2 Supplementary Table 2. Deregulated miRNAs identified by the microarray in PAH rats.
