# Supplementary material for: Upregulation of miR-335-5p Contributes to Right Ventricular Remodeling via Calumenin in Pulmonary Arterial Hypertension
Source: Biomed Res Int. 2022 Oct 4;2022:9294148. doi: 10.1155/2022/9294148 (PMC9557250; doi:10.1155/2022/9294148)
Supplement: Supplementary 1 — Supplementary Table 1. Primer sequences used for quantitative RT-qPCR. [file 9294148.f1.docx]

| Target gene | Forward primers (5’-3’) | Reverse primers (5’-3’) |
| --- | --- | --- |
| miR-212-3p (rat) | CATCGCTAACAGTCTCCAGTCA | CACTTCCTCAGCACTTGTTGGTAT |
| miR-1247-3p (rat) | CAGTGCATAGCCACGTAACG | GCCGTAACCACTAATCCG |
| miR-335-5p (rat) | CGTCCTCGTCAAGAGCAATAAC | TATGCTTGTTCTCGTCTCTGTGTC |
| miR-3592 (rat) | ACACTCCAGCTGGGAAGTGTTGTCCGTGA | TGGTGTCGTGGAGTCG |
| miR-382-3p (rat) | CAGGCAATCATTCACGGACA | TATGCTTGTTCACGACACCTTCAC |
| miR-411-3p (rat) | TTCGGTCCTATGTAACACGGTC | TAAGGTTCTTCACGACTGGTTCAC |
| U6 (rat) | CAGCACATATACTAAAATTGGAACG | ACGAATTTGCGTGTCATCC |
| CALU（rat） | TACCGCTTTTCTGCACCCTGAG | AGGCCTCCCCGAAATCTGTG |
| GAPDH (rat) | GGTGGACCTCATGGCCTACA | CTCTCTTGCTCTCAGTATCCTTGCT |
| ANP (rat) | GGCGGCACTTAGCTCCCTCTC | GGGCTCCAATCCTGTCAATCCTAC |
| β-MHC (rat) | CGCTCCACGCACCCTCACTTTGT | CAGCCCCGCCTTGAAGAACACCT |
| CALU（mouse） | AATGCTGATGGGTTCATTGATCT | GGTTCTTATCTCGAAACTCCACG |
| GAPDH (mouse) | AGGTCGGTGTGAACGGATTTG | TGTAGACCATGTAGTTGAGGTCA |
| ANP (mouse) | GCTTCCAGGCCATATTGGAG | GGGGGCATGACCTCATCTT |
| β-MHC (mouse) | ACTGTCAACACTAAGAGGGTCA | TTGGATGATTTGATCTTCCAGGG |

**Supplementary Table 1. Primer sequences used for quantitative RT-qPCR.**
