# Supplementary material for: Upregulation of miR-335-5p Contributes to Right Ventricular Remodeling via Calumenin in Pulmonary Arterial Hypertension
Source: Biomed Res Int. 2022 Oct 4;2022:9294148. doi: 10.1155/2022/9294148 (PMC9557250; doi:10.1155/2022/9294148)
Supplement: Supplementary 2 — Supplementary Table 2. Deregulated miRNAs identified by the microarray in PAH rats. [file 9294148.f2.docx]

| miRNA name | Regulation direction | Fold change (log2) | P value |
| --- | --- | --- | --- |
| miR-212-3p | Up | 3.1293 | 0.00000 |
| miR-1247-3p | Up | 2.0949 | 0.00027 |
| miR-335-5p | Up | 1.8111 | 0.00011 |
| miR-21-5p | Up | 1.8030 | 0.00000 |
| miR-199a-5p | Up | 1.7428 | 0.00007 |
| miR-214-3p | Up | 1.7343 | 0.00000 |
| miR-184 | Up | 1.6984 | 0.00130 |
| miR-877 | Up | 1.6516 | 0.00034 |
| miR-31a-5p | Up | 1.6285 | 0.00087 |
| miR-132-3p | Up | 1.6243 | 0.00110 |
| miR-3592 | Down | -1.6679 | 0.00062 |
| miR-382-3p | Down | -1.6679 | 0.00062 |
| miR-411-3p | Down | -1.5511 | 0.00757 |
| miR-370-3p | Down | -1.5434 | 0.01015 |
| miR-323-3p | Down | -1.5063 | 0.00165 |
| miR-329-5p | Down | -1.5056 | 0.00647 |
| miR-409b | Down | -1.4981 | 0.00016 |
| miR-201-5p | Down | -1.4951 | 0.00003 |
| miR-409a-3p | Down | -1.4834 | 0.00016 |
| miR-708-3p | Down | -1.4439 | 0.00016 |

**Supplementary Table 2. Deregulated miRNAs identified by the microarray in PAH rats**
